# Effects of a quercetin-rich onion skin extract on 24 h ambulatory blood pressure and endothelial function in overweight-to-obese patients with (pre-)hypertension: a randomised double-blinded placebo-controlled cross-over trial

**DOI:** 10.1017/S0007114515002950

**Published:** 2015-09-02

**Authors:** Verena Brüll, Constanze Burak, Birgit Stoffel-Wagner, Siegfried Wolffram, Georg Nickenig, Cornelius Müller, Peter Langguth, Birgit Alteheld, Rolf Fimmers, Stefanie Naaf, Benno F. Zimmermann, Peter Stehle, Sarah Egert

**Affiliations:** 1 Department of Nutrition and Food Sciences, Nutritional Physiology, University of Bonn, 53115 Bonn, Germany; 2 Institute of Clinical Chemistry and Clinical Pharmacology, University Hospital Bonn, 53127 Bonn, Germany; 3 Institute of Animal Nutrition and Physiology, Christian-Albrechts-University Kiel, 24118 Kiel, Germany; 4 Department of Cardiology, Angiology and Pneumology, University Hospital Bonn, 53127 Bonn, Germany; 5 Department of Biopharmaceutics and Pharmaceutical Technology, Institute of Pharmacy and Biochemistry, Johannes Gutenberg University Mainz, 55099 Mainz, Germany; 6 Institute of Medical Biometry, Informatics and Epidemiology, University Hospital Bonn, 53127 Bonn, Germany; 7 Institut Prof. Dr. Georg Kurz GmbH, 50933 Köln, Germany; 8 Department of Nutrition and Food Sciences, Food Technology and Biotechnology, University of Bonn, 53117 Bonn, Germany

**Keywords:** Quercetin, Blood pressure, Endothelial function, Hypertension, Cardiovascular diseases

## Abstract

The polyphenol quercetin may prevent CVD due to its antihypertensive and vasorelaxant properties. We investigated the effects of quercetin after regular intake on blood pressure (BP) in overweight-to-obese patients with pre-hypertension and stage I hypertension. In addition, the potential mechanisms responsible for the hypothesised effect of quercetin on BP were explored. Subjects (*n* 70) were randomised to receive 162 mg/d quercetin from onion skin extract powder or placebo in a double-blinded, placebo-controlled cross-over trial with 6-week treatment periods separated by a 6-week washout period. Before and after the intervention, ambulatory blood pressure (ABP) and office BP were measured; urine and blood samples were collected; and endothelial function was measured by EndoPAT technology. In the total group, quercetin did not significantly affect 24 h ABP parameters and office BP. In the subgroup of hypertensives, quercetin decreased 24 h systolic BP by −3·6 mmHg (*P*=0·022) when compared with placebo (mean treatment difference, −3·9 mmHg; *P*=0·049). In addition, quercetin significantly decreased day-time and night-time systolic BP in hypertensives, but without a significant effect in inter-group comparison. In the total group and also in the subgroup of hypertensives, vasoactive biomarkers including endothelin-1, soluble endothelial-derived adhesion molecules, asymmetric dimethylarginine, angiotensin-converting enzyme activity, endothelial function, parameters of oxidation, inflammation, lipid and glucose metabolism were not affected by quercetin. In conclusion, supplementation with 162 mg/d quercetin from onion skin extract lowers ABP in patients with hypertension, suggesting a cardioprotective effect of quercetin. The mechanisms responsible for the BP-lowering effect remain unclear.

Quercetin (3,3′,4′,5,7-pentahydroxyflavone) is one of the predominant flavonoids, ubiquitously distributed in (edible) plants, and one of the most potent antioxidants of plant origin^(^ ^1^ ^)^. Rich sources of dietary quercetin are onions, kale, unpeeled apples, berries, citrus fruits and tea (*Camellia sinensis*). In Western populations, crude estimates of mean dietary intake appear to be 10–30 mg/d^(2^ ^,^ ^3^ ^)^. As demonstrated in cohort studies, dietary intake of flavonoids in general and of quercetin in particular is associated with a decreased risk for CVD^(^ ^2^ ^,^ ^4^ ^)^. Although the physiological mechanisms accounting for this benefit remain incompletely defined, animal studies and human intervention studies have identified many relevant effects – for example, supplementation of quercetin may reduce platelet aggregation^(^ ^5^ ^,^ ^6^ ^)^ and plasma concentrations of atherogenic oxidised LDL (oxLDL)^(^ ^7^ ^)^. *In vitro* studies suggest that high concentrations of quercetin (>1 µm) have anti-inflammatory effects^(^ ^8^ ^,^ ^9^ ^)^. Studies in animal models (e.g. obese Zucker rats) suggest beneficial effects of quercetin on obesity-associated metabolic disorders including insulin resistance and dyslipidaemia^(^ ^10^ ^–^ ^12^ ^)^. We recently showed that in patients with high CVD risk phenotype, chronic supplementation with a supra-nutritional dose of 150 mg/d quercetin significantly reduced systolic blood pressure (SBP)^(^ ^7^ ^)^. Similar findings have been reported by Edwards *et al.* ^(^ ^13^ ^)^ in hypertensive patients and, recently, by Zahedi *et al.* ^(^ ^14^ ^)^ in women with type 2 diabetes mellitus, although with pharmacological quercetin doses (500 and 730 mg/d). Although several pathways have been suggested, the mechanisms by which quercetin possibly affects blood pressure (BP) are not well-understood. These pathways include (i) improvement of vascular function in an endothelium-dependent or endothelium-independent manner, (ii) decrease in oxidative stress and/or (iii) interference with the renin–angiotensin–aldosterone system^(^ ^15^ ^)^.

One limitation in the interpretation of the BP-lowering effect of chronic quercetin supplementation is that all human studies published to date, including our own trial^(^ ^7^ ^)^, only measured the office (clinic) BP in the resting state (typically in the morning while fasting) and did not integrate an ambulatory blood pressure (ABP) monitoring. ABP monitoring is considered the gold standard for BP measurement, compared with clinic BP measurements, as it is superior in terms of reliability and validity^(^ ^16^ ^,^ ^17^ ^)^. The superior predictive power of ABP monitoring is probably not only due to its higher number of readings, which increases the reliability of the measurement, but also due to its ability to capture the impact of stressors and other environmental factors that occur in daily life and are likely to affect BP^(^ ^16^ ^)^. To the best of our knowledge, no previous study has examined the effects of quercetin on 24 h ABP profiles thus far. Therefore, the aim of the present double-blinded, placebo-controlled cross-over trial was to systematically investigate the effects of quercetin on arterial BP (office BP and 24 h ABP profiles) in adults with pre-hypertension and stage I hypertension, and to explore mechanisms involved in the BP-lowering efficacy of quercetin. Furthermore, effects of quercetin on lipid and glucose metabolism were investigated.

## Methods

### Subjects

Overweight-to-obese subjects were recruited from the community of the city of Bonn, Germany, via public postings, flyers and advertisements in the local newspaper. From a total of 500 interested subjects, 154 individuals aged 25–65 years with a BMI of 25–35 kg/m^2^ attended a screening, which included physical assessments (body height and weight, resting BP, heart rate, waist and hip circumference), clinical assessments (liver function, serum lipids and lipoproteins, glucose and uric acid, haematology, high-sensitive C-reactive protein (hs-CRP)), medical history and a dietary questionnaire.

Participants were included if they had the following traits of the metabolic syndrome: (i) central obesity (waist circumference ≥94 cm for men and ≥80 cm for women); (ii) pre-hypertension (≥120–139 mmHg SBP and/or ≥80–89 mmHg diastolic blood pressure (DBP)) or stage I hypertension (≥140–159 mmHg SBP and/or ≥90–99 mmHg DBP); (iii) dyslipidaemia (fasting serum TAG concentrations ≥1·7 mmol/l and/or serum concentrations of HDL-cholesterol <1·0 mmol/l for men and <1·3 mmol/l for women) and/or a proinflammatory state (hs-CRP≥2 mg/l)^(^ ^18^ ^,^ ^19^ ^)^. Main exclusion criteria were as follows: smoking, diagnosed type 2 diabetes mellitus, liver, gastrointestinal or diagnosed inflammatory diseases, a history of cardiovascular events, untreated thyroid dysfunction, cancer, recent major surgery or illness, pregnancy or breast feeding, alcohol abuse, consumption of polyphenol-rich supplements, participation in a weight loss programme and Raynaud’s syndrome.

**Fig. 1:**
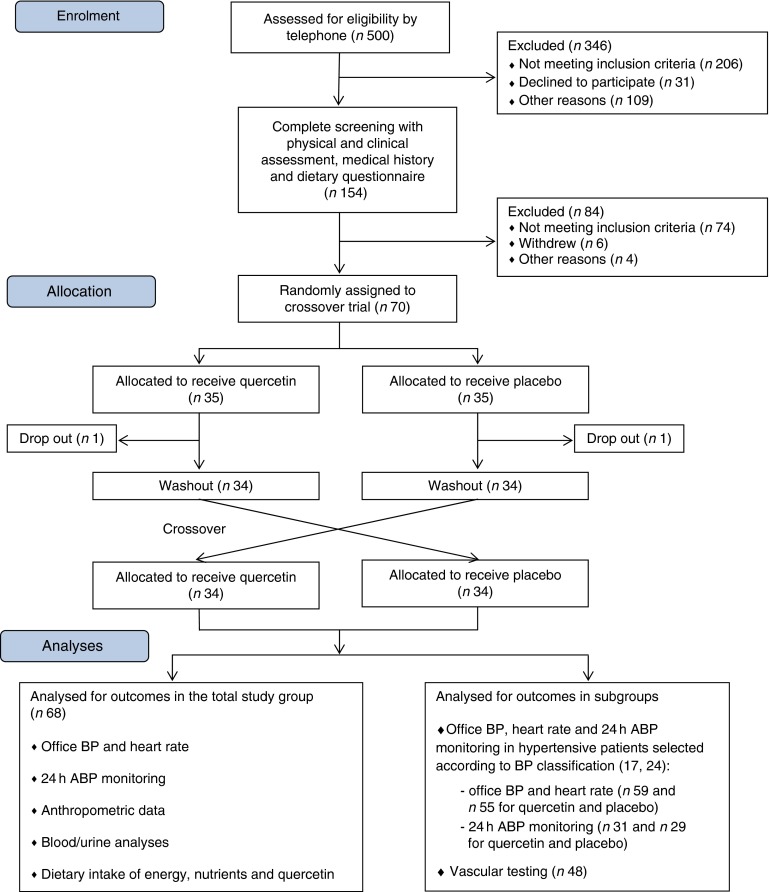
Flow diagram of participants. ABP, ambulatory blood pressure; BP, blood pressure.

A total of seventy subjects (thirty-five male, thirty-five female) were included into the study. During the first intervention period, two subjects dropped out for personal reasons. Only data from the remaining sixty-eight subjects (thirty-four male, thirty-four female), who completed the entire intervention study, were included in the analysis and are subsequently reported. The participant flow from the initial screening to final analysis is shown in Fig. 1.

The study protocol was explained in detail to all the participants, who gave their written informed consent at the beginning of the study. The study protocol was approved by the ethics committee of the Medical Faculty of the Rheinische Friedrich-Wilhelms-Universität Bonn, Germany, and was in accordance with the Helsinki declaration. The trial was registered at www.germanctr.de/ and http://apps.who.int/trialsearch/ as DRKS00000555.

The subjects were instructed to maintain their habitual diet, physical activity levels, lifestyle factors and body weight. Participants taking oral contraceptives (*n* 4 women) or antihypertensive (*n* 12) or thyroid drugs (*n* 9) were instructed to continue taking their medication without changes.

### Study design

This study was a double-blinded, randomised, placebo-controlled cross-over trial with 6-week treatment periods separated by a 6-week washout period. Subjects were instructed to take a total of three capsules per d, one capsule with each principal meal. Two kinds of hard gelatin capsules – quercetin and placebo – were manufactured at the Institute of Pharmacy and Biochemistry, Johannes Gutenberg University, Mainz. Quercetin capsules were filled with onion skin extract powder (132 mg/capsule); placebo capsules contained mannitol (approximately 170 mg/capsule). For the production of the onion skin extract powder, onion skins were washed with water and then extracted using ethanol. Thereafter, the extraction solvent was removed by evaporation. The resulting suspension was decanted and subsequently vacuum-dried. The flavonoid contents of the onion skin extract and of quercetin capsules were determined by HPLC with diode-array detection. The identity of the flavonoids was confirmed by MS/MS. The results of quantification are shown in Table 1. The quercetin content of the onion skin extract powder (*Allium cepa* L.; Rudolf Wild GmbH & Company KG) was 41·25 % (dry mass 95·94 %). Each quercetin capsule contained 54 mg quercetin. Hard gelatin capsules (Coni-Snap^®^) size 0 were supplied by Capsugel. Quercetin and placebo capsules were identical in shape and taste. Capsule filling was carried out using a Dott Bonapace semi-automatic capsule filling machine. Mannitol was obtained from Fagron. Quality was checked by determining the homogeneity of weight distributions of a sample of twenty randomised capsules taken from each batch. Furthermore, the microbiological burden of the capsules was determined following manufacture and before release. Primary packaging was into blister packages. The primary investigators, all study personnel, and all the participants were blinded to the treatments. A quercetin dosage of 162 mg per d (three capsules) was selected to represent the 10- to 15-fold of the estimated mean daily quercetin intake in Germany^(^ ^20^ ^)^ and other European populations^(^ ^21^ ^)^. Plasma kinetics of this quercetin dosage after bolus intake was examined previously^(^ ^22^ ^,^ ^23^ ^)^.

**Table 1:** Flavonoid analysis in onion skin extract powder* (Mean values and standard deviations)

	% (g/100 g)	
Compound	Mean	sd
Quercetin	44·2	0·15
Quercetin dihexoside	0·04523	0·00027
Quercetin hexoside 1	0·1557	0·0072
Quercetin hexoside 2	1·79	0·017
Methylquercetin hexoside	0·0481	0·00030
Kaempferol	0·122	0·0017
Methylquercetin	0·0740	0·00033

* Analyses were conducted in duplicate.

Subjects were assigned to quercetin or placebo treatment according to a block-wise randomisation scheme. Separate computer-generated randomisation schedules for men and women were created to stratify subjects by sex with an attempt to achieve a distribution between men and women of 50:50 in each treatment group. Randomisation, allocation to either one of the capsules and capsule handling were carried out by an independent researcher (B. A.). Capsules were handed out on days 0 and 21 of each treatment period with a surplus of 20 %. Leftovers and empty blisters were collected on days 21 and 42. Compliance was monitored by determining quercetin plasma concentrations after the treatment periods (see below), by counting capsules at the end of the study and by instructing subjects to document capsule consumption, potential observed side-effects, deviations from their normal physical activity and any other observations considered relevant in a study diary.

Subjects were instructed to keep 3-d food records at the beginning and at the end of each treatment period. Each record represented the food and beverage intake of 2 weekdays and 1 weekend day. These dietary records were used to calculate the habitual dietary intake of energy, nutrients and quercetin.

Sample size was calculated based on office BP data from our previous trial^(^ ^7^ ^,^ ^24^ ^)^ and expected changes in SBP. The calculation revealed that forty-nine subjects had to complete the study to reach a 90 % power with a significance level of 0·01 to detect a 3 mmHg difference in BP between the quercetin and placebo groups (sd of the within-subject was different from the SBP, 3·8 mmHg). With the assumption of a 30 % dropout rate, we aimed to randomly assign approximately seventy participants.

The measurements of 24 h ABP, office BP, heart rate, vascular testing and anthropometric measurements were conducted at the beginning and at the end of the two intervention periods.

### Measurements

#### Office blood pressure and heart rate

Measurements of office BP and heart rate were obtained using an automatic BP measurement device (boso carat professional, Bosch+Sohn) under standardised conditions according to the recommendations of the American Heart Association Council on High Blood Pressure Research^(^ ^25^ ^)^. Each participant sat quietly for 5–10 min, after which their arm was placed at heart level and SBP and DBP were measured at least twice in 3- to 5-min intervals. If BP measurements varied by 10 mmHg, an additional measurement was performed. The accumulated measurements were then averaged to determine overall SBP and DBP. The mean arterial pressure (MAP) was calculated as follows: (DBP+1/3 (SBP−DBP)).

#### 24 h ambulatory blood pressure monitoring

The 24 h ABP and heart rate recordings were taken every 15 min between 06.00 and 22.00 hours (day-time) and every 30 min between 22.00 and 06.00 hours (night-time) using an ABP monitor (Spacelabs monitor type 90207) on the non-dominant arm. On the day of measurement, subjects were instructed to maintain their habitual activity level and to refrain from strenuous exercise. We assessed the following BP parameters: 24 h, day-time and night-time SBP, DBP, MAP, heart rate and the nocturnal dip in SBP and DBP.

#### Vascular testing

For vascular testing, we used the noninvasive peripheral arterial tonometry (PAT) technology to assess the reactive hyperaemia index (RHI) and the augmentation index (AI) using the EndoPAT plethysmographic device (Endo-PAT2000). Details have been described elsewhere^(^ ^26^ ^)^. In brief, the PAT signal indicates changes of the peripheral arterial tone in peripheral arterial beds by recording the arterial pulsatile volume changes from the fingertip. For that purpose, we placed a pair of plethysmographic biosensors on both index fingers and a BP cuff on the upper arm of the study arm (left arm), whereas the right arm served as the control arm (without a cuff). The recording of the PAT signal started after a resting period of at least 15 min with a 1 min standby-test period. If the signal recording was free from interferences, the test period started with a 5 min baseline recording of the pulse wave amplitude. Subsequently, the BP cuff was inflated for 5 min to supra-systolic values to induce ischaemia whereupon the cuff was deflated. This resulted in reactive hyperaemia while further recording for 5 min. PAT signals were analysed with automated software (Itamar Medical), and RHI was calculated as the ratio of the average PAT signal amplitude over a 1 min period starting 1 min after cuff-deflation divided by the average PAT signal amplitude over a 3·5 min period at the baseline recording followed by normalisation of the values from the study arm to the control arm. The RHI correlates with brachial artery flow-mediated dilatation^(^ ^27^ ^)^ and is significantly influenced by nitric oxide (NO)^(^ ^28^ ^)^. A lower RHI was found in subjects with coronary endothelial dysfunction^(^ ^29^ ^)^ and in the presence of CVD risk factors^(^ ^30^ ^)^.

#### Anthropometrics

Body height was determined on a stadiometer to the nearest 0·1 cm. Body weight was determined to the nearest 100 g. Waist circumference was measured midway between the lowest rib and the ilial crest, while the subject was at minimal respiration. Hip circumference was measured at the height of trochanteres majores. Body composition was measured by bioelectrical impedance analysis (Nutrigard-M, Multi Frequency Phase-Sensitive Bioelectrical Impedance Analyzer, Data Input). Fat-free mass (FFM) was calculated in accordance with Sun *et al.* ^(^ ^31^ ^)^; fat mass was calculated by subtracting FFM from body weight.

### Blood/urine sample processing and analysis

Fasting venous blood samples were collected on the first and the last visit of each intervention period between 06.30 and 09.00 hours under standardised conditions. The subjects were instructed to abstain for 24 h from alcoholic beverages and were told not to engage in strenuous exercise on the day before blood sampling. The last capsule was taken in the evening before blood sampling. Blood was drawn into tubes containing EDTA, lithium heparin, fluoride or a coagulation activator (Sarstedt). Plasma/serum was obtained by centrifugation at 3000 ***g*** for 15 min at 8°C. Plasma/serum aliquots were immediately frozen in cryovials and stored at −80°C until analysis. All the laboratory measurements were performed without knowledge of the treatment. All serum and plasma samples of one subject were analysed in the same assay run.

Serum concentration of total cholesterol was measured using polychromatic endpoint-measurement, whereas serum concentrations of LDL-cholesterol, HDL-cholesterol, TAG and plasma concentrations of glucose were measured using bichromatic endpoint-measurement with a Dimension Vista 1500 analyser (Siemens Healthcare Diagnostics). Serum concentrations of apo B, A1 and hs-CRP were determined using nephelometric methods with a Dimension Vista 1500 analyser.

Serum insulin concentration was measured using a chemiluminescent–immunometric assay with the Immulite 2000 analyser (Siemens Healthcare Diagnostics). Insulin resistance was measured using the homoeostatic model assessment (HOMA) and calculated as the product of the fasting plasma insulin concentration (in μU/ml) and the fasting plasma glucose concentration (in mmol/l), divided by 22·5^(^ ^32^ ^)^. HbA_1c_ was measured using an HPLC-method with a Variant II analyser (Bio-Rad Laboratories). Serum concentrations of angiotensin-converting enzyme (ACE) were measured using a photometric method (Buehlmann Laboratories) with a Dimension Vista 1500 analyser.

Plasma ADMA (Immundiagnostik), plasma oxLDL (Immundiagnostik), serum endothelin-1 and serum-soluble adhesion molecules E-selectin, soluble intercellular adhesion molecule 1 (sICAM-1) and soluble vascular cell adhesion molecule 1 (sVCAM-1) (R&D systems) were determined in duplicate using commercially available enzyme-linked immunoassay kits according to the manufacturer’s instructions and quality controls. Plasma concentration of l-arginine was determined using reversed-phase HPLC as described previously^(^ ^33^ ^)^.

Analyses of plasma concentrations of quercetin, its monomethylated derivatives tamarixetin (4′-*O*-methyl quercetin) and isorhamnetin (3′-*O*-methyl quercetin) as well as of the dehydroxylated quercetin metabolite kaempferol were carried out using HPLC with fluorescence detection as described previously^(^ ^34^ ^)^. All the samples were treated enzymatically with *β*-glucuronidase/sulphatase before the extraction of the flavonols. Total plasma flavonols were calculated as follows: total flavonols (nmol/l)=quercetin (nmol/l)+kaempferol (nmol/l)+isorhamnetin (nmol/l)+tamarixetin (nmol/l).

First morning urine samples were collected on the first and the last visit of the intervention periods, 0·002 % of butylated hydroxytoluene was added and the urine was frozen at −80 °C until analysis. From these urine samples, 8-iso-PG F2*α* (8-iso-PGF2*α*) and 2,3-dinor-15-F2t-IsoP were measured by UHPLC-MS/MS. Urinary creatinine levels were determined using a bichromatic kinetic method (Jaffé method).

### Self-reported dietary intake of energy, nutrients and quercetin

The self-reported intakes of energy, macronutrients, dietary fibre and antioxidant pro-vitamins/vitamins were calculated using the computer-based nutrient calculation programme EBISpro (University of Hohenheim) based on the German Nutrient Database Bundeslebensmittelschlüssel (Max Rubner-Institute). The quercetin intake was estimated using the USDA flavonoid database^(^ ^35^ ^)^.

### Statistical analyses

All the statistical analyses were performed using IBM SPSS statistical software package (version 20). Differences between sexes at screening were tested using the unpaired Student’s *t* test or the Mann–Whitney *U* test. Baseline values were compared between groups using paired Student’s *t* tests or Wilcoxon signed-rank tests. Intra-group (baseline *v.* endpoint) and inter-group comparisons (changes during quercetin *v.* changes during placebo treatment) of normally distributed data were performed using paired Student’s *t* tests. Intra-group and inter-group comparisons of data that were not normally distributed, which was mainly the case for serum TAG, plasma glucose, serum insulin, HOMA IR index, plasma oxLDL, serum hs-CRP and urinary IsoP, were conducted by Wilcoxon signed-rank tests (for details, see footnotes in Tables).

Interaction effects between the stage of hypertension and treatment assignment were tested using a univariate ANOVA with the respective variables as fixed factors. In all cases, a value for *P*≤0·05 (two-sided) was taken to indicate significant effects. Pearson’s correlation coefficient was used to assess relationships between BP variables (SBP and DBP) and biomarkers of inflammation/endothelial function and also between BP variables and plasma flavonols. A test for carry-over effects according to Kenward and Jones^(^ ^36^ ^)^ was used. No carry-over effects between the two treatment periods could be observed. All the analyses are presented on a per-protocol basis. For all sixty-eight participants, complete data sets were available.

All data were analysed for the whole study group (*n* 68) and also for the subgroup of patients with hypertension. Office BP data were classified according to Chobanian *et al.* ^(^ ^18^ ^)^. Classification of ABP data was conducted as described by Pickering *et al.* ^(^ ^25^ ^)^ (for details see footnotes in Table 4).

**Table 4:** Measurements of the 24 h ambulatory blood pressure and office blood pressure in the subgroup of hypertensive patients during the 6-week dietary supplementation with quercetin or placebo* (Mean values and standard deviations)

	Quercetin							Placebo										
	Baseline		Endpoint		Mean change		*P* intra-group	Baseline		Endpoint		Mean change		*P* intra-group	*P* inter-group	Treatment difference	
	Mean	sd	Mean	sd	Mean	sd	comparison	Mean	sd	Mean	sd	Mean	sd	comparison	comparison	Mean	sd
24 h ABP monitoring†	(*n* 31)							(*n* 29)										
Systolic BP (mmHg)	141·5	7·3	137·9	7·3	−3·6	8·2	0·022	139·7	5·3	140·2	6·4	+0·5	6·4	0·690	0·049	−3·9	11·1
Diastolic BP (mmHg)	88·7	6·6	86·6	5·9	−2·1	6·2	0·074	86·6	6·1	86·9	7·3	+0·3	3·9	0·680	0·124	−2·8	8·3
MAP (mmHg)	106·4	6·5	103·7	5·9	−2·8	7·3	0·043	104·4	5·4	104·7	6·5	+0·3	4·4	0·710	0·151	−3·0	9·7
Heart rate (beats per min)	76·1	8·3	77·9	8·6	+1·8	5·5	0·081	76·6	10·8	77·5	9·7	+0·9	5·9	0·445	0·435	+1·3	7·9
Nocturnal dip in systolic BP (%)	12·1	6·5	12	6·6	+0·3	5·4	0·758	9·6	5·8	12·5	5·9	+2·9	7·1	0·035	0·142	−2·8	8·7
Nocturnal dip in diastolic BP (%)	16·1	7·7	16·9	7·6	+0·8	5·8	0·477	13·9	7·6	17·3	7·8	+3·3	8·7	0·051	0·213	−2·5	9·5
Day-time (06.00–22.00 hours)‡	(*n* 27)							(*n* 23)										
Systolic BP (mmHg)	146·4	6·7	141·8	7·7	−4·6	9·0	0·014	144·5	4·4	144·6	6·3	+0·1	5·9	0·964	0·109	−4·9	12·1
Diastolic BP (mmHg)	92·9	5·8	90·4	6·3	−2·5	6·9	0·070	90·3	6·5	90·9	7·4	+0·5	2·9	0·399	0·111	−3·6	9·0
MAP (mmHg)	110·8	5·9	107·54	6·5	−3·3	8·0	0·042	108·4	5·2	108·8	6·5	+0·4	3·6	0·644	0·110	−4·2	10·6
Heart rate (beats per min)	79·2	9·0	80·8	9·1	+1·6	6·3	0·211	79·0	9·9	80·5	9·3	+1·4	6·7	0·311	0·793	−0·6	9·1
Night-time (22.00–06.00 hours)§	(*n* 21)							(*n* 27)										
Systolic BP (mmHg)	134·7	8·4	128·1	9·6	−6·6	9·9	0·007	131·1	6·7	126·1	9·8	−5·0	10·6	0·021	0·667	−2·2	18·8
Diastolic BP (mmHg)	80·6	9·4	75·8	8·1	−4·9	7·2	0·006	78·8	6·5	75·7	8·2	−3·1	7·6	0·045	0·289	−3·5	11·8
MAP (mmHg)	99·7	8·5	93·0	7·9	−6·7	8·3	0·001	96·6	6·9	92·9	8·6	−3·8	8·9	0·037	0·345	−3·8	14·4
Heart rate (beats per min)	68·7	8·5	71·6	9·3	+2·9	6·3	0·049	69·5	11·0	67·5	8·5	−2·0	8·7	0·235	0·127	+4·3	9·8
Office BP‖	(*n* 59)							(*n* 55)										
Systolic BP (mmHg)	147·1	16·2	149·3	17·0	+2·2	10·9	0·131	147·8	12·7	147·2	16·9	−0·6	11·6	0·685	0·311	+2·3	16·3
Diastolic BP (mmHg)	100·3	10·6	98·6	9·6	−1·7	8·7	0·138	100·7	8·0	98·8	10·2	−1·8	7·5	0·072	0·972	−0·1	12·4
MAP (mmHg)	115·9	11·7	115·5	11·4	−0·4	8·7	0·718	116·4	8·6	114·9	11·8	−1·4	8·0	0·186	0·676	+0·7	12·5
Heart rate (beats per min)	65·0	7·6	65·9	8·0	+0·8	5·5	0·256	64·4	8·9	65·8	7·9	+1·3	6·1	0·109	0·575	−0·6	8·2

ABP, Ambulatory blood pressure; BP, blood pressure; MAP, mean arterial pressure.

* The two groups did not differ significantly with regard to any of the variables at baseline (paired Student’s *t* tests or Wilcoxon signed-rank tests). All the subjects participated in both treatments. There are different n values for quercetin and placebo treatment because the initial blood pressure of the participants at the beginning of the quercetin and placebo treatment period slightly differed. In some cases, this physiological difference led to a different blood pressure classification.

† Systolic BP>135 and/or diastolic BP>85 mmHg (according to reference 25).

‡ Systolic BP>140 and/or diastolic BP>90 mmHg (according to reference 25).

§ Systolic BP>125 and/or diastolic BP>75 mmHg (according to reference 25).

‖ Stage 1 hypertensive: systolic BP≥140 and/or diastolic BP≥90 mmHg (according to reference 18).

## Results

### Subject characteristics at screening, compliance and dietary intake

Characteristics of the participants at screening are presented in Table 2. As planned, all subjects were overweight (43 %) or obese (57 %), had a visceral fat distribution and were pre-hypertensive or stage 1 hypertensive. We observed sex differences with respect to body height, body weight, waist and hip circumference, waist:hip ratio and fasting serum concentrations of TAG, total cholesterol, HDL-cholesterol and plasma glucose (Table 2).

**Table 2:** Subject characteristics and blood parameters at screening (Mean values and standard deviations)

	Total (*n* 68)		Women (*n* 34)		Men (*n* 34)		
	Mean	sd	Mean	sd	Mean	sd	Women *v.* men (*P*)
Age (years)	47·4	10·5	48·2	10·4	46·6	10·6	0·396
Body height (cm)	173·8	9·6	168·2	9·0	179·3	6·6	<0·0001
Body weight (kg)	94·4	16·1	88·8	15·5	99·9	14·9	0·021
BMI (kg/m^2^)	31·1	3·4	31·3	3·6	31·0	3·2	0·677
Overweight (%)	43		50		35		
Obese (%)	57		50		65		
Waist circumference (cm)	103·9	10·4	99·0	8·5	108·7	10·0	0·008
Hip circumference (cm)	111·5	8·7	115·3	8·8	107·7	6·7	0·001
Waist:hip ratio	0·93	0·09	0·86	0·05	1·01	0·05	<0·0001
Systolic BP (mmHg)	144·4	12·6	141·7	13·4	147·2	11·3	0·077
Diastolic BP (mmHg)	94·2	8·4	94·2	8·1	94·3	8·8	0·687
Heart rate (beats per min)	73·0	9·6	74·1	9·1	71·8	10·2	0·496
Serum TAG (mmol/l)	1·98	1·04	1·71	0·82	2·25	1·17	0·014
Serum total cholesterol (mmol/l)	5·69	1·15	5·97	1·21	5·41	1·03	0·042
Serum HDL-cholesterol (mmol/l)	1·46	0·34	1·64	0·33	1·27	0·23	<0·0001
Serum LDL-cholesterol (mmol/l)	3·59	0·93	3·72	0·95	3·45	0·89	0·232
Plasma glucose (mmol/l)	5·19	0·55	5·00	0·42	5·38	0·60	0·013
Serum insulin (pmol/l)	67·9	43·4	57·9	24·5	77·2	54·4	0·082
Serum hs-CRP (mg/l)	3·19	6·04	2·93	3·22	3·45	7·98	0·690

BP, blood pressure; hs-CRP, high-sensitive C-reactive protein.

**Fig. 2:**
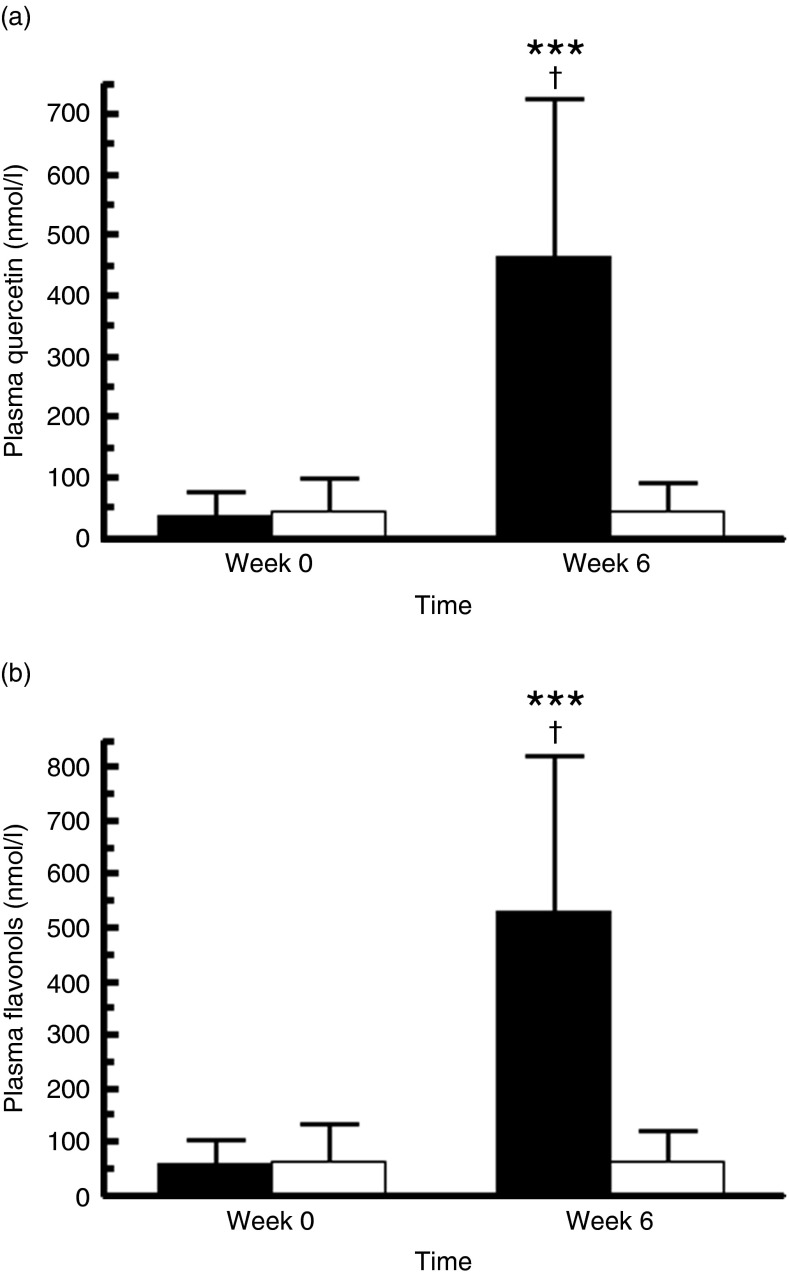
Fasting plasma concentrations of quercetin (a) (*n* 68) and total flavonols (b) (*n* 68) before and after the 6-week supplementation with quercetin (162 mg/d; ■) or placebo (□). Values are means and standard deviations represented by vertical bars. *** Mean value was significantly different from baseline (*P*<0·0001; intra-group comparison). † Change during quercetin treatment was significantly different from change during placebo treatment (*P*<0·0001; inter-group comparison). Total plasma flavonols were calculated as follows: total flavonols (nmol/l)=quercetin (nmol/l)+kaempferol (nmol/l)+isorhamnetin (nmol/l)+tamarixetin (nmol/l). The two groups did not differ significantly with regard to plasma concentrations of quercetin and total flavonol at baseline (Wilcoxon signed-rank tests).

Count of returned capsules indicated an almost full compliance of 98·2 (sd 2·6) % and 98·0 (sd 4·1) % during quercetin and placebo consumption, respectively. Compliance to quercetin supplementation was objectively confirmed by a marked increase in plasma concentrations of quercetin and total flavonols by 1149·6 % (*P*<0·0001) and 828·6 % (*P*<0·0001), respectively (Fig. 2(a) and (b)). The increase was measurable in all patients receiving quercetin. In addition, plasma concentrations of kaempferol, isorhamnetin and tamarixetin significantly increased after quercetin but not after placebo supplementation (data not shown). There was a high inter-individual variation in plasma quercetin concentrations already at baseline (range for all study subjects: 0·2–330·5 nmol/l) and after quercetin supplementation (99·1–1313·1 nmol/l).

Analyses of 3-d dietary records indicated no significant inter-group and intra-group differences in mean daily intakes of energy, protein, carbohydrates, total fat, fatty acids, cholesterol, antioxidants (e.g. vitamin E, vitamin C), dietary fibre and quercetin (data not presented in Tables). Dietary quercetin intake was 11·3 (sd 11·9) mg/d and 9·0 (sd 8·1) mg/d at the beginning and at the end of the quercetin treatment, respectively, and 11·7 (sd 11·2) mg/d and 9·2 (sd 8·2) mg/d at the beginning and at the end of the placebo treatment, respectively. Main dietary quercetin sources were onions, apples and tea.

### Body weight, waist and hip circumference, body composition and potential side-effects

Quercetin supplementation did not significantly affect body weight, waist and hip circumference, relative fat mass or fat-free mass (data not shown). Participants did not report any side-effects, neither during quercetin nor during placebo treatment.

### Ambulatory blood pressure monitoring and office blood pressure

In the entire study group (*n* 68), quercetin and placebo supplements did not significantly affect mean 24 h, day-time and night-time ABP parameters (SBP, DBP, MAP, nocturnal dip in SBP and DBP). In addition, office BP (SBP and DBP, MAP) was not significantly changed by quercetin or placebo treatment as analysed for the entire study group (Table 3). Resting SBP at baseline and the change in SBP from baseline to after intervention were significantly correlated, with those individuals with higher baseline SBP demonstrating greater reductions in SBP in response to the quercetin treatment (*r* −0·508, *P*<0·0001; *n* 68). We also found a significant interaction effect between stage of hypertension and treatment assignment for the decreases in mean 24 h SBP (*P*=0·008) and in day-time SBP (*P*=0·024) but not for the decrease in night-time SBP (*P*=0·568) (data not shown). There were also significant interaction effects between stage of hypertension and treatment assignment for decreases in mean 24 h MAP (*P*=0·014) and in day-time MAP (*P*=0·032) but not for decreases in night-time MAP (*P*=0·176) (data not shown).

**Table 3:** Measurements of the 24 h ambulatory blood pressure and office blood pressure in the total study group during the 6-week dietary supplementation with quercetin or placebo* (Mean values and standard deviations)

	Quercetin (*n* 68)							Placebo (*n* 68)										
	Baseline		Endpoint		Mean change		*P* intra-group	Baseline		Endpoint		Mean change		*P* intra-group	*P* inter-group	Treatment difference	
	Mean	sd	Mean	sd	Mean	sd	comparison	Mean	sd	Mean	sd	Mean	sd	comparison	comparison	Mean	sd
24 h ABP monitoring																	
Systolic BP (mmHg)	133·3	10·2	132·4	9·0	−0·9	7·3	0·329	132·5	8·4	132·4	9·6	−0·1	6·0	0·848	0·460	−0·8	9·1
Diastolic BP (mmHg)	82·0	8·5	81·4	7·6	−0·7	5·3	0·304	81·4	7·1	80·9	8·5	−0·4	4·5	0·425	0·711	−0·2	6·8
MAP (mmHg)	99·5	8·7	98·6	7·5	−0·9	6·1	0·227	98·8	7·0	98·4	8·3	−0·4	4·7	0·520	0·977	−0·5	7·7
Heart rate (beats per min)	74·3	8·2	76·3	8·2	+2·0	5·3	0·003	74·5	9·8	75·1	8·7	+0·7	5·9	0·372	0·189	+1·3	8·1
Nocturnal dip in systolic BP (%)	11·0	6·6	11·3	5·7	+0·3	6·1	0·726	10·7	5·6	12·0	4·8	+1·3	6·5	0·105	0·368	−1·1	9·5
Nocturnal dip in diastolic BP (%)	15·9	7·1	16·9	6·8	+1·0	6·4	0·217	16·0	6·7	17·5	6·2	+1·5	7·3	0·101	0·669	−0·6	10·6
Day-time (06.00–22.00 hours)																	
Systolic BP (mmHg)	136·6	10·6	135·7	9·4	−0·9	7·6	0·347	135·5	8·5	136·1	9·7	+0·6	6·2	0·440	0·172	−1·6	9·2
Diastolic BP (mmHg)	85·0	8·7	84·5	7·9	−0·5	5·6	0·457	84·3	7·2	84·2	8·6	−0·0	4·7	0·932	0·538	−0·5	6·9
MAP (mmHg)	102·5	8·8	101·8	7·9	−0·8	6·4	0·315	101·4	7·1	101·7	8·4	+0·3	4·9	0·640	0·257	−1·1	7·8
Heart rate (beats per min)	76·2	8·7	78·2	8·6	+1·9	5·6	0·006	76·7	10·5	77·5	9·2	+0·7	6·4	0·367	0·202	+1·3	8·4
Night-time (22.00–06.00 hours)																	
Systolic BP (mmHg)	121·4	11·7	120·3	10·4	−1·1	9·0	0·322	120·9	10·5	119·6	9·8	−1·3	9·1	0·238	0·921	+0·2	14·0
Diastolic BP (mmHg)	71·4	9·2	70·1	8·0	−1·3	6·4	0·106	70·8	8·7	69·4	8·5	−1·4	6·4	0·085	0·946	+0·8	9·9
MAP (mmHg)	88·5	9·9	87·0	8·0	−1·5	7·4	0·093	88·1	9·1	86·7	8·5	−1·4	7·5	0·139	0·911	−0·2	11·5
Heart rate (beats per min)	66·9	8·7	69·2	8·8	+2·4	6·6	0·004	67·4	9·8	68·1	9·1	+0·7	7·3	0·459	0·203	+1·7	10·7
Office BP																	
Systolic BP (mmHg)	144·4	16·8	146·9	17·2	+2·5	10·7	0·063	143·9	14·4	143·8	17·4	−0·1	10·9	0·937	0·171	+2·6	15·3
Diastolic BP (mmHg)	98·2	11·3	97·2	9·7	−1·0	8·5	0·338	97·3	10·0	96·1	11·0	−1·2	7·3	0·166	0·863	+0·2	11·5
MAP (mmHg)	113·6	12·5	113·8	11·6	+0·2	8·5	0·879	112·8	10·7	112·0	12·6	−0·9	7·7	0·361	0·472	+1·0	11·6
Heart rate (beats per min)	64·6	7·9	65·7	8·3	+1·1	5·5	0·112	63·9	9·1	65·6	8·0	+1·6	6·4	0·040	0·598	−0·5	8·2

ABP, ambulatory blood pressure; BP, blood pressure; MAP, mean arterial pressure.

* The two groups did not differ significantly with regard to any of the variables at baseline (paired Student’s *t* tests or Wilcoxon signed-rank tests).

Effects of supplementation with quercetin or placebo on ABP profiles and office BP in the subgroup of hypertensive patients are presented in Table 4. Note that there are different n values for quercetin and placebo treatment because the initial BP of the participants at the beginning of the quercetin and placebo period slightly differed. In some cases, this physiological difference led to a different BP classification. When compared with placebo (treatment difference, −3·9 (sd 11·1) mmHg; *P*=0·049, Table 4), quercetin significantly decreased mean 24 h SBP by −3·6 (sd 8·2) mmHg (*P*=0·022) from baseline in the subgroup of stage 1 hypertensive subjects (*n* 31). Changes in day-time SBP and night-time SBP during quercetin treatment were not significantly different from placebo treatment. However, quercetin significantly decreased day-time SBP by −4·6 (sd 9·0) mmHg (*P*=0·014) and night-time SBP by −6·6 (sd 9·9) mmHg (*P*=0·007) (Table 4). In addition, changes in 24 h MAP, day-time MAP and night-time MAP during quercetin did not differ significantly from placebo. However, quercetin significantly reduced mean 24 h MAP, day-time MAP and night-time MAP from baseline by −2·8 (sd 7·3) mmHg (*P*=0·043), by −3·3 (sd 8·0) mmHg (*P*=0·042) and by −6·7 (sd 8·3) mmHg (*P*=0·001), respectively (Table 4).

Treatment with quercetin did not significantly change the nocturnal dip in SBP and DBP. In addition, systolic and diastolic office BP were not significantly changed by quercetin or placebo treatment in hypertensive patients (Table 4).

### Serum lipids, lipoproteins, apolipoproteins, glucose and insulin

No significant inter-group differences were found for the effects of quercetin or placebo on serum lipids, lipoproteins, apolipoproteins, glucose and insulin. Furthermore, quercetin supplementation did not significantly affect fasting serum total cholesterol, LDL-cholesterol, HDL-cholesterol, TAG, apo B and A1, insulin, plasma glucose and HbA_1c_ neither in the total study group nor in the subgroup of hypertensive patients (intra-group comparisons, Table 5).

**Table 5:** Fasting lipids, lipoproteins, apolipoproteins, insulin, glucose, HbA_1c_ and HOMA IR Index in the total study group (*n* 68) during the 6-week dietary supplementation with quercetin or placebo* (Mean values and standard deviations)

	Quercetin (*n* 68)							Placebo (*n* 68)										
	Baseline		Endpoint		Mean change		*P* value intra-group	Baseline		Endpoint		Mean change		*P* value intra-group	*P* value inter-group	Treatment difference	
	Mean	sd	Mean	sd	Mean	sd	comparison	Mean	sd	Mean	sd	Mean	sd	comparison	comparison	Mean	sd
Serum TAG (mmol/l)	1·81	1·09	1·83	1·37	+0·03	0·98	0·848	1·76	1·25	1·72	1·37	−0·04	0·68	0·241	0·628	+0·07	1·18
Serum total cholesterol (mmol/l)	5·44	1·12	5·38	1·12	−0·06	0·58	0·395	5·45	1·13	5·38	1·09	−0·08	0·55	0·257	0·874	+0·02	0·89
Serum HDL-cholesterol (mmol/l)	1·39	0·38	1·36	0·37	−0·03	0·19	0·238	1·38	0·34	1·38	0·35	−0·00	0·14	0·929	0·406	−0·03	0·25
Serum LDL-cholesterol (mmol/l)	3·45	0·90	3·41	0·91	−0·04	0·43	0·459	3·52	0·95	3·38	0·88	−0·14	0·41	0·008	0·193	+0·10	0·63
Serum apo B (g/l)	1·05	0·26	1·04	0·25	−0·02	0·12	0·257	1·05	0·26	1·04	0·25	−0·02	0·12	0·233	0·864	+0·00	0·18
Serum apo A1 (g/l)	1·63	0·28	1·60	0·26	−0·03	0·17	0·128	1·62	0·26	1·60	0·27	−0·02	0·13	0·240	0·824	−0·01	0·23
Plasma glucose (mmol/l)	5·08	0·68	5·11	0·58	+0·02	0·47	0·526	5·18	0·63	5·13	0·71	−0·03	0·51	0·671	0·400	+0·07	0·71
Serum insulin (pmol/l)	47·82	33·62	46·60	35·86	−1·22	24·48	0·649	51·15	38·75	46·39	38·85	−4·75	26·14	0·079	0·197	+3·53	39·16
HOMA IR index	1·57	1·34	1·51	1·32	−0·05	0·92	0·911	1·72	1·42	1·52	1·41	−0·17	0·99	0·071	0·226	+0·13	1·41
HbA_1c_ (%)	5·66	0·43	5·68	0·45	+0·02	0·19	0·366	5·70	0·42	5·66	0·38	−0·03	0·18	0·146	0·105	+0·05	0·27

HOMA IR, homoeostasis model assessment of insulin resistance.

* The two groups did not differ significantly with regard to any of the variables at baseline (paired Student’s *t* tests or Wilcoxon signed-rank tests).

### Soluble adhesion molecules, asymmetric dimethylarginine, angiotensin-converting enzyme, C-reactive protein, endothelial function and oxidation

For the total study group, quercetin supplementation did not significantly affect fasting serum concentrations of endothelin-1, sE-selectin and sVCAM-1 (Table 6). When compared with placebo, quercetin did not significantly affect serum sICAM-1 (treatment difference, −5·3 (sd 30·7) ng/ml, *P*=0·219; Table 6). However, quercetin decreased fasting serum sICAM-1 from baseline by −8·2 (sd 17·2) ng/ml (intra-group comparison, *P*<0·001). Neither quercetin nor placebo significantly altered fasting plasma concentrations of the endogenous NO synthase inhibitor ADMA, the corresponding ratio of l-arginine to ADMA (l-arginine:ADMA), serum ACE and serum CRP. Quercetin and placebo did not significantly affect endothelial function (RHI and AI) (Table 6). In addition, plasma oxLDL and urinary excretion of 8-iso-PGF2*α* and 2,3-dinor-15-F2t-IsoP were not significantly changed by quercetin or placebo treatment in the total study group (Table 6).

**Table 6:** Adhesion molecules, parameters of oxidation and inflammation and vascular testing in the total study group (*n* 68) during the 6-week supplementation with quercetin or placebo* (Mean values and standard deviations)

	Quercetin (*n* 68)							Placebo (*n* 68)										
	Baseline		Endpoint		Mean change		*P* intra-group	Baseline		Endpoint		Mean change		*P* intra-group	*P* inter-group	Treatment difference	
	Mean	sd	Mean	sd	Mean	sd	comparison	Mean	sd	Mean	sd	Mean	sd	comparison	comparison	Mean	sd
Serum endothelin-1 (pg/ml)	2·22	0·74	2·25	0·74	+0·03	0·46	0·639	2·09	0·71	2·25	0·78	+0·16	0·52	0·014	0·129	−0·13	0·71
Serum sE-selektin (ng/ml)	36·2	13·6	37·1	13·7	+0·9	5·1	0·144	36·7	13·8	36·9	14·0	+0·2	4·8	0·712	0·441	+0·7	7·3
Serum sVCAM-1 (ng/ml)	581·0	161·9	571·6	143·4	−9·3	99·7	0·444	572·5	152·3	595·4	155·8	+22·9	99·9	0·063	0·055	−32·2	135·8
Serum sICAM-1 (ng/ml)	205·7	54·2	197·5	48·1	−8·2	17·2	<0·001	202·7	53·0	199·8	51·6	−2·9	26·7	0·378	0·219	−5·3	30·7
Plasma oxLDL (ng/ml)	408·2	348·8	406·0	300·8	−2·2	202·7	0·859	371·9	236·4	386·9	221·1	+15·1	114·7	0·342	0·352	−17·3	235·7
Plasma ADMA (µmol/l)	0·57	0·16	0·59	0·15	+0·01	0·15	0·530	0·60	0·21	0·58	0·15	−0·02	0·18	0·439	0·266	+0·03	0·21
l-Arginine:ADMA-ratio	146·6	66·8	147·8	55·3	+1·2	73·6	0·896	141·1	57·8	144·8	51·8	+3·7	54·8	0·577	0·824	−2·6	94·4
Serum ACE (U/l)	38·2	19·2	38·4	20·2	+0·2	7·0	0·800	38·3	19·5	39·3	19·8	+1·1	6·8	0·198	0·718	−0·9	10·9
Serum hs-CRP (mg/l)	2·20	1·99	2·21	2·05	+0·02	2·17	0·904	2·24	2·33	2·81	3·61	+0·58	3·33	0·448	0·283	−0·56	4·04
Urinary 8-iso-PGF2*α*/creatinine (pg/mg)	198·7	160·5	216·8	156·7	+5·8	177·8	0·183	236·6	293·6	187·1	132·9	−56·2	316·3	0·848	0·552	–17·9	300·6
Urinary 2,3-dinor-15-F2t-IsoP/creatinine (pg/mg)	644·8	342·4	645·7	393·6	+0·9	257·8	0·608	664·5	396·0	669·1	370·4	+4·3	259·8	0·788	0·984	−1·0	390·1
RHI (EndoPAT)	1·90	0·60	1·89	0·56	−0·01	0·55	0·911	1·91	0·57	1·88	0·48	−0·04	0·66	0·650	0·762	+0·03	0·69
AI (EndoPAT)†	8·35	21·89	8·44	21·91	+0·08	10·59	0·957	6·63	17·35	7·44	20·98	+0·81	10·04	0·578	0·733	−0·73	14·71

ACE, angiotensin-converting enzyme; ADMA, asymmetric dimethylarginine; AI, augmentation index; hs-CRP, high-sensitive C-reactive protein; IsoP, isoprostanes; oxLDL, oxidised LDL; RHI, reactive hyperaemia index; sICAM-1, soluble intercellular adhesion molecule 1; sVCAM-1, soluble vascular cell adhesion molecule 1.

* The two groups did not differ significantly with regard to any of the variables at baseline (paired Student’s *t* tests or Wilcoxon signed-rank tests). EndoPAT data were obtained in a subgroup of forty-eight participants.

† Corrected for heart rate and normed to 75 bpm.

In the subgroup of hypertensive patients, neither quercetin nor placebo significantly affected serum endothelin-1, sE-selectin, sVCAM-1, plasma ADMA, serum ACE and CRP (data not in Tables). In addition, plasma oxLDL and urinary F2-IsoP were not significantly changed by quercetin or placebo treatment in the subgroup of hypertensives (data not in Tables). In the subgroup of hypertensive patients, quercetin and placebo significantly decreased serum sICAM-1 concentrations from baseline to a similar extent (quercetin, −9·3 (sd 14·8) ng/ml, *P*=0·002; placebo, −8·6 (sd 15·9) ng/ml, *P*=0·007; treatment difference, −3·5 (sd 17·5) ng/ml, *P*=0·584) (data not in Tables).

### Correlations between blood pressure and biomarkers of inflammation/endothelial function

In the total study group and also in the subgroup of hypertensive patients, baseline SBP and DBP did not significantly correlate with baseline biomarkers of inflammation and endothelial function (CRP, endothelin-1, sE-selectin, sVCAM-1, sICAM-1, ADMA, RHI and AI). In addition, changes in BP variables did not significantly correlate with changes in biomarkers of inflammation and endothelial function, neither in the total study group nor in the subgroup of hypertensives (data not shown).

## Discussion

The aim of this double-blinded, placebo-controlled, cross-over intervention study was to systematically investigate the effects of quercetin on arterial BP (primary variable), endothelial function and further CVD risk markers in overweight-to-obese individuals with hypertension. Our major finding was that quercetin supplementation for 6 weeks in a supra-nutritional dosage significantly reduced 24 h systolic ABP in hypertensive participants. In contrast to our hypotheses, we observed no effect of quercetin supplementation on secondary variables such as endothelial function including RHI, sE-selectin, sVCAM-1, ADMA and endothelin-1. In addition, no effects on parameters of inflammation, oxidative stress and lipid and glucose metabolism were observed. We used a moderate supra-nutritional but non-pharmacological dose of quercetin from onion skin extract, as these data should provide a rational basis for the development of functional foods.

### Blood pressure

Quercetin significantly reduced the mean 24 h SBP by 3·6 (sd 8·2) mmHg in hypertensive participants, but not in pre-hypertensive individuals, suggesting that a threshold of elevated BP might be required to detect a BP-lowering effect of quercetin. The significant interaction effect between stage of hypertension and treatment assignment confirms this. In contrast to our previous trial^(^ ^7^ ^)^, we found no significant effects of chronic quercetin supplementation on office SBP. At first glance, this finding is surprising. Our study design (e.g. quercetin dose, intervention period) was similar to our earlier study. In addition, our participants (pre-/hypertensive patients with abdominal fat distribution) and sample size were well chosen in consideration with the primary objective of the trial. In accordance with our previous study^(^ ^7^ ^)^, we observed a relatively high inter-individual variation in BP-lowering effects of quercetin. In both studies, only 44 to 51 % of the participants showed a decrease in systolic office BP, despite the fact that compliance with supplementation in both trials was very high (94–98 %). Compliance was confirmed by a marked increase in plasma quercetin concentrations (final concentrations of both trials, 0·3–0·45 µmol/l) and also in the monomethylated derivatives isorhamnetin and tamarixetin. However, in both trials, changes in BP were not significantly correlated with plasma total quercetin, isorhamnetin and/or tamarixetin. These findings indicate that, in humans, the BP-lowering effects of chronic quercetin supplementation are difficult to predict. In addition, it is not possible to deduce a minimum plasma concentration or quercetin dose required for a specific biological activity. In this regard, it is very likely that the plasma concentration of total quercetin does not necessarily reflect the concentration at the target sites. Similar high inter-individual variance in BP-lowering effects of quercetin can be observed in the study of Edwards *et al.* ^(^ ^13^ ^)^ in human subjects treated with pharmacological doses of 730 mg/d quercetin for 4 weeks. The authors illustrated individual subject responses during each supplementation phase (placebo, quercetin) in their publication; however, they did not discuss these results.

Overall, the quercetin-induced reductions in SBP observed in the present trial and previously (range −2·6 to −8·8 mmHg)^(^ ^7^ ^,^ ^13^ ^,^ ^14^ ^,^ ^24^ ^)^ are similar to those experienced following current recommended lifestyle modifications to reduce elevated BP (e.g. reducing sodium intake and body weight, increasing physical activity)^(^ ^37^ ^,^ ^38^ ^)^. A 3·6 mmHg decrease in SBP as observed in the present trial would be clinically meaningful when considered at the population level, particularly in view of the large population of people with pre-hypertension and stage I hypertension^(^ ^39^ ^)^. In addition, similar effects have been found for other dietary flavonoids, especially for flavonols from cocoa products^(^ ^40^ ^,^ ^41^ ^)^.

### Endothelial function, inflammation and oxidation

The first mechanism of action we explored was that quercetin-induced reductions in BP are secondary to an improvement in vascular endothelial function. Rationale for this hypotheses was based on data from (i) normotensive humans wherein acute administration of 200 mg quercetin increased plasma quercetin and NO metabolite concentrations while decreasing endothelin-1^(^ ^42^ ^)^, (ii) hypertensive rats in which quercetin attenuated hypertension and/or vascular dysfunction in a NO-dependent manner^(^ ^43^ ^–^ ^45^ ^)^ and (iii) *in vitro* studies wherein quercetin decreased cellular production of endothelin-1^(^ ^46^ ^,^ ^47^ ^)^ and endothelial-derived adhesion molecules^(^ ^48^ ^,^ ^49^ ^)^. In the present study, biomarkers of endothelial function (plasma endothelin-1, sE-selectin, sVCAM-1, ADMA, RHI and AI) were unaffected by the quercetin treatment. Therefore, these markers cannot explain a possible relationship between quercetin and BP. However, in view of the only modest decrease in SBP, our study was probably not suited to demonstrate small changes in these markers. In addition, baseline PAT indices and endothelial-derived adhesion molecules did not correlate with BP, emphasising that factors regulating endothelial function differ at least in part from those regulating BP.

In contrast to previous *in vitro* ^(^ ^8^ ^,^ ^9^ ^)^ and *in vivo* results obtained in animal models^(^ ^49^ ^–^ ^51^ ^)^ showing an anti-inflammatory effect of quercetin, quercetin supplementation did not reduce serum concentrations of CRP. This may, in part, be explained by the duration of the intervention and/or the quercetin dosage and/or the low-grade inflammatory state of our participants, which were insufficient to elicit measurable effects. In agreement with this finding and consistent with earlier studies in humans^(^ ^13^ ^,^ ^23^ ^,^ ^52^ ^)^, no changes in biomarkers of *in vivo* lipid peroxidation (plasma oxLDL and urinary IsoP) were observed following quercetin administration. This may be attributed to an already sufficient antioxidant defence status of our hypertensive subjects. All participants had an adequate dietary intake of essential antioxidants (vitamins E, C, *β*-carotene). In addition, they did not report smoking or excessive physical exercise.

### Angiotensin-converting enzyme

The second mechanism we hypothesised was that chronic quercetin supplementation decreased arterial BP in hypertensive patients by decreasing ACE activity. Rationale for this hypotheses was based on experimental studies demonstrating that quercetin inhibits ACE *in vitro* ^(^ ^53^ ^)^ and on animal-based evidence showing a decrease in ACE activity after quercetin treatment in rats^(^ ^54^ ^,^ ^55^ ^)^. For example, Mackraj *et al.* ^(^ ^54^ ^)^ compared the long-term antihypertensive effects of captopril (ACE inhibitor) with those of quercetin in Dahl salt-sensitive rats that were given daily injections of captopril, quercetin or vehicle. Although BP increased in vehicle-treated Dahl rats, it was significantly decreased compared with baseline in both quercetin- and captopril-treated groups. The decrease in BP occurred in parallel with the down-regulation of the angiotensin-I receptor in the kidney, increased urine volume and increased urinary sodium excretion, thus providing a potential mechanism for the long-term BP-lowering effects of quercetin^(^ ^15^ ^)^. However, although chronic quercetin administration decreased 24 h ABP in our hypertensive patients, we found no decrease in plasma ACE activity. Our results are in accordance with data recently reported by Larson *et al.* ^(^ ^56^ ^)^ showing that acute, quercetin-induced reductions in BP in hypertensive individuals were not secondary to a reduced ACE activity.

### Lipid and glucose metabolism

In the present study, chronic quercetin supplementation for 6 weeks did not affect fasting serum concentrations of total cholesterol, LDL-cholesterol and HDL-cholesterol, TAG and apolipoproteins, supporting previous data in metabolically healthy participants^(^ ^23^ ^,^ ^57^ ^)^, in overweight-to-obese patients with metabolic syndrome traits^(^ ^7^ ^)^, in men with different apoE isoforms^(^ ^52^ ^)^ and in women with type 2 diabetes mellitus^(^ ^14^ ^)^. In contrast, Lee *et al.* ^(^ ^58^ ^)^ found that supplementation with quercetin (100 mg/d) for 10 weeks significantly reduced fasting serum concentrations of total cholesterol and LDL-cholesterol and significantly increased serum HDL-cholesterol in male smokers. In addition, supplements of quercetin-rich red grape juice^(^ ^59^ ^)^ and grape powder^(^ ^60^ ^)^ favourably influenced plasma lipid profiles in human subjects. These inconclusive results may be attributed to the duration of quercetin administration, the baseline characteristics of the study participants and the dosage of the ingested quercetin. Experimental studies in rat liver cells demonstrated that quercetin at high concentrations (e.g. 25 µmol/l) may be involved in lipid metabolism by reducing hepatic fatty acid, TAG and cholesterol synthesis^(^ ^61^ ^,^ ^62^ ^)^.

In accordance with previous human studies^(^ ^7^ ^,^ ^13^ ^,^ ^24^ ^,^ ^52^ ^,^ ^60^ ^)^, quercetin did not affect fasting plasma glucose, serum insulin, HOMA IR or HbA_1c_ in our hypertensive patients, although Lee *et al.* ^(^ ^58^ ^)^ demonstrated a slight decrease (−3·9 %) in fasting serum glucose after quercetin treatment. Our results are in contrast to experimental reports demonstrating the hypoglycaemic effects of high quercetin doses (≥10 mg/kg of body weight) in animal models (e.g. obese Zucker Rats)^(^ ^10^ ^–^ ^12^ ^)^.

### Strengths and limitations

The major strengths of our study are the double-blinded placebo-controlled cross-over design, the relatively large sample size, the high compliance to treatment, the low dropout rate and the examination of numerous markers of vascular function. However, this study also has a few potential limitations. First, we administered a quercetin-rich onion skin extract instead of pure quercetin (quercetin dihydrate) as we did in our previous trials^(^ ^7^ ^,^ ^23^ ^,^ ^24^ ^)^. Onion skins are rich in quercetin aglycone^(^ ^63^ ^)^, and the bioavailability of quercetin from capsules filled with onion skin extract powder was shown to be significantly higher than that from capsules filled with quercetin dihydrate (C Burak, V Brüll, P Langguth, BF Zimmermann, B Stoffel-Wagner, P Stehle, S Wolffram, S Egert, unpublished results). Thus, we decided to use a quercetin-rich onion skin extract for the present trial. We characterised the polyphenol spectrum of the extract (Table 1), but we cannot exclude that other unknown components in the onion skin extract may have influenced our findings. Thus, strictly, the conclusion of our study is only true for quercetin-rich onion skin extract but not for pure quercetin. Second, we conducted an explorative data analysis. All parameters were analysed for the whole study group and also for the subgroup of patients with hypertension. Due to the multiple test situations, we cannot fully exclude that the significant effect on 24 h SBP in the subgroup of hypertensives was a chance finding. On the other hand, we do not think that corrections for multiple testing are meaningful in the context of explorative data analysis^(^ ^64^ ^)^.

### Conclusion

Regular ingestion of a supra-nutritional dose of 162 mg/d quercetin from onion skin extract did not affect BP and endothelial function in the whole study population of pre-hypertensive and stage 1 hypertensive overweight-to-obese subjects. In the subgroup of hypertensives, quercetin was capable of decreasing 24 h SBP, suggesting a cardioprotective effect of quercetin, but without effects on mechanistic parameters. Thus, the mechanisms responsible for the BP-lowering effect of quercetin remain elusive.
